# Identification of a broad-spectrum lytic *Myoviridae* bacteriophage using multidrug resistant *Salmonella* isolates from pig slaughterhouses as the indicator and its application in combating *Salmonella* infections

**DOI:** 10.1186/s12917-022-03372-8

**Published:** 2022-07-12

**Authors:** Mengfei Zhao, Rui Xie, Shuang Wang, Xi Huang, Hao Yang, Wenqing Wu, Lin Lin, Hongjian Chen, Jie Fan, Lin Hua, Wan Liang, Jianmin Zhang, Xiangru Wang, Huanchun Chen, Zhong Peng, Bin Wu

**Affiliations:** 1 State Key Laboratory of Agricultural Microbiology, College of Veterinary Medicine, Huazhong Agricultural University, Wuhan, 430070 China; 2 Key Laboratory of Preventive Veterinary Medicine in Hubei Province, The Cooperative Innovation Centre for Sustainable Pig Production, Huazhong Agricultural University, Wuhan, 430070 China; 3 Present address: Hubei Jin Xu Agricultural Development Limited by Share Ltd., Wuhan, China; 4 National and Regional Joint Engineering Laboratory for Medicament of Zoonoses Prevention and Control, College of Veterinary Medicine, South China Agricultural University, Guangzhou, China; 5 Hubei Hongshan Laboratory, Wuhan, China

**Keywords:** *Salmonella*, Antimicrobial resistance, Prevalence, Slaughterhouse, Lytic bacteriophage, Application

## Abstract

**Background:**

*Salmonella* is a leading foodborne and zoonotic pathogen, and is widely distributed in different nodes of the pork supply chain. In recent years, the increasing prevalence of antimicrobial resistant *Salmonella* poses a threat to global public health. The purpose of this study is to the prevalence of antimicrobial resistant *Salmonella* in pig slaughterhouses in Hubei Province in China, and explore the effect of using lytic bacteriophages fighting against antimicrobial resistant *Salmonella*.

**Results:**

We collected a total of 1289 samples including anal swabs of pigs (862/1289), environmental swabs (204/1289), carcass surface swabs (36/1289) and environmental agar plates (187/1289) from eleven slaughterhouses in seven cities in Hubei Province and recovered 106 *Salmonella* isolates. Antimicrobial susceptibility testing revealed that these isolates showed a high rate of antimicrobial resistance; over 99.06% (105/106) of them were multidrug resistant. To combat these drug resistant *Salmonella*, we isolated 37 lytic phages using 106 isolates as indicator bacteria. One of them, designated ph 2–2, which belonged to the *Myoviridae* family, displayed good capacity to kill *Salmonella* under different adverse conditions (exposure to different temperatures, pHs, UV, and/or 75% ethanol) and had a wide lytic spectrum. Evaluation in mouse models showed that ph 2–2 was safe and saved 80% (administrated by gavage) and 100% (administrated through intraperitoneal injection) mice from infections caused by *Salmonella Typhimurium*.

**Conclusions:**

The data presented herein demonstrated that *Salmonella* contamination remains a problem in some pig slaughter houses in China and *Salmonella* isolates recovered in slaughter houses displayed a high rate of antimicrobial resistance. In addition, broad-spectrum lytic bacteriophages may represent a good candidate for the development of anti-antimicrobial resistant *Salmonella* agents.

**Supplementary Information:**

The online version contains supplementary material available at 10.1186/s12917-022-03372-8.

## Background

*Salmonella* is a leading cause of diarrhea and an important foodborne pathogen. The Centers for Disease Control and Prevention (CDC) estimates *Salmonella* bacteria cause about 1.35 million infections, 26,500 hospitalizations, and 420 deaths in the United States every year [1]. In China, a laboratory-based surveillance revealed 3% (*n* = 662) of *Salmonella enterica* infections in 23,140 stool specimens in 126 hospitals in 44 cities and counties from eight provinces [2]. Another analysis on 29,210 diarrheal patients in the outpatient department of a hospital in China between 1998 and 2013 has identified *Salmonella* as the third-most frequent cause of diarrhea from 1998 to 2006, as the second-most frequent cause from 2006 to 2010, and as the most frequent cause from 2011 to 2013 [3]. To date, more than 2500 serovars have been described for *Salmonella*, but only less than 100 serotypes account for most infections in humans [4]. Of particular note is serovar Typhimurium, which is responsible for the majority of *Salmonella* infection cases worldwide [5]. Ecologically, *Salmonella* bacteria are widely distributed in animals, particularly in food animals such as poultry, pigs, and cattle, and the inhabitant environment of humans and animals [6]. Investigation of contaminated food and drinking water has been recognized as a main reason for *Salmonella* infections in humans [1].

The antimicrobial resistance (AMR) condition of *Salmonella* has also raised a global concern in recent years [7]. Over the past few decades, the prevalence of antibiotic resistant *Salmonella* has increased in many regions of the world, including the developed world such as Australia, the United States, and the European Union [7, 8]. The rapid emergence and dissemination of antibiotic resistant *Salmonella*, in particular those resistance to the last-resort antibiotics such as colistin, carbapenems, and/or tigecycline, may raise the difficulty of treatment or lead to the treatment failure in both human and veterinary medicine [9–11]. From this point, seeking alternative options combating against antibiotic resistant *Salmonella* is important and necessary. Since their discovery in 1915, lytic bacteriophages (or phages) have been proposed as promising therapeutic tools for infections caused by antibiotic-resistant bacteria due to their inherent capacity to kill pathogens [12]. Recently, phages have achieved a great success in treating patients infected by multidrug resistant bacteria [13]. In agriculture and food industry, the Food and Drug Administration (FDA) have approved the use of phages for *Salmonella* control in poultry, and against *E. coli* in red meat [14]. In this study, we investigated the prevalence and AMR profile of *Salmonella* in pig slaughterhouses in Hubei Province, China. By using those *Salmonella* isolates recovered as indicator bacteria, we isolated many lytic *Salmonella* phages and established a *Salmonella* phage library. One phage isolate showed a broad-spectrum of killing antibiotic resistant *Salmonella* strains belonging to different serotypes. This phage also exhibited good effect on control *Salmonella* infection in mouse models.

## Results

### Antimicrobial susceptibility of *Salmonella* isolates from slaughterhouses in Hubei Province

**Fig. 1:**
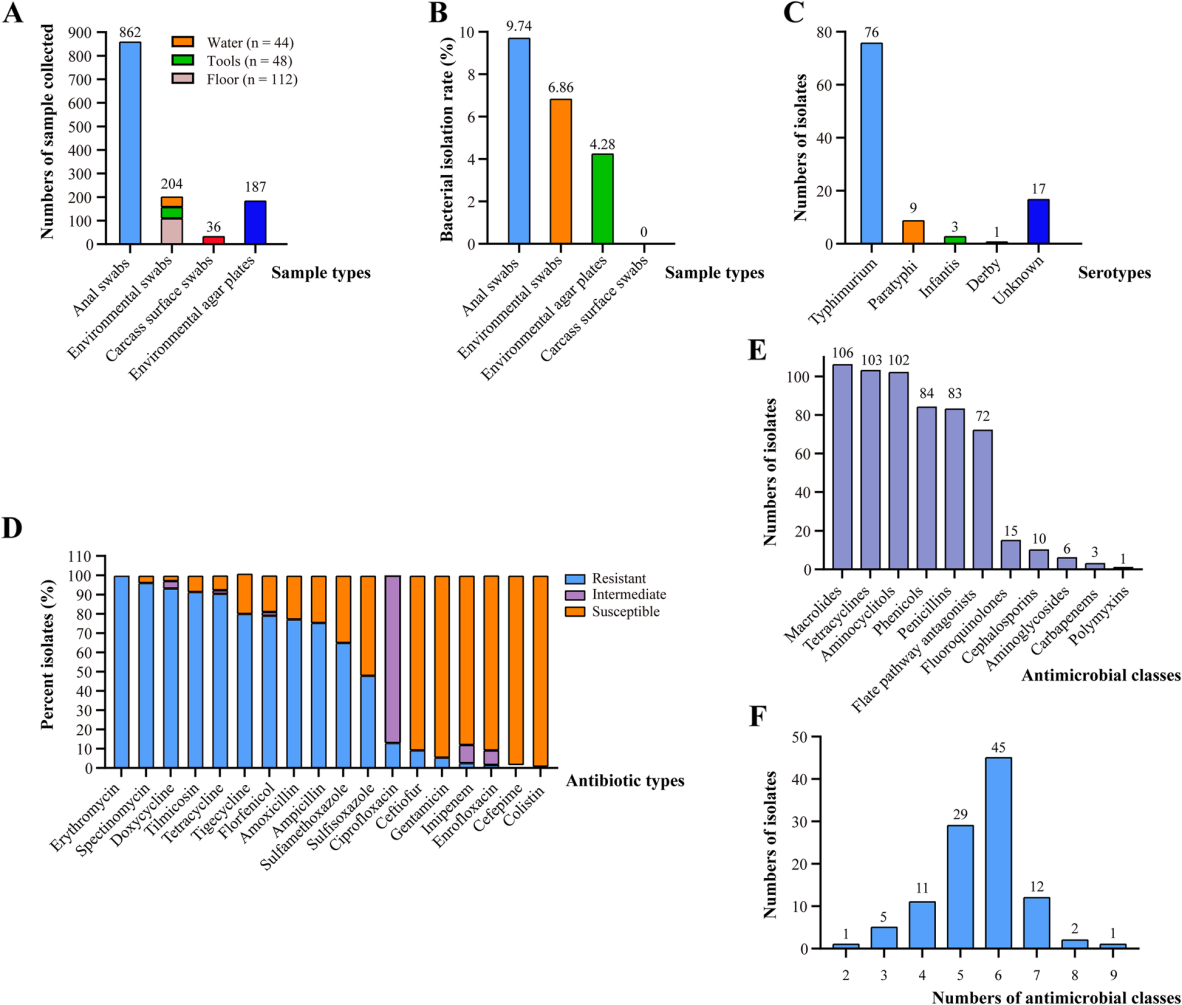
Isolation and antimicrobial resistant phenotypes of *Salmonella* from pig slaughter houses in Hubei Province in China. **A** A column chart showing the distribution of different types of samples collected for *Salmonella* isolation; **B** A column chart showing the isolation rates of *Salmonella* from different types of samples; **C** A column chart showing the distribution of different *Salmonella* serovars; **D** A column chart showing the percent isolates of *Salmonella* with different phenotypes against different antibiotics; **E** A column chart showing the numbers of *Salmonella* isolates with resistant phenotypes to different antimicrobial classes; **F** A column chart showing the numbers of *Salmonella* isolates resisting different numbers of antimicrobial classes

Between July 1, 2020 and June 30, 2021, we collected a total of 1289 samples including anal swabs of pigs (862/1289), environmental swabs (204/1289), carcass surface swabs (36/1289) and environmental agar plates (187/1289) from eleven slaughterhouses in seven cities in Hubei Province in China and recovered 106 *Salmonella* isolates from these samples (Fig. 1A). The total isolation rates of *Salmonella* from different types of samples from the eleven slaughterhouses ranged from 0 (0/36) to 9.74% (84/862) (Fig. 1B). Determination of serovars demonstrated four types of serovars, and 71.70% (76/106) of the isolates belonged to *Salmonella Typhimurium* (Fig. 1C).

Antimicrobial susceptibility testing (AST) revealed that over 90% of the isolates were resistant to erythromycin (100%, 106/106), spectinomycin (96.23%, 102/106), doxycycline (93.40%, 99/106), tilmicosin (91.51%, 97/106), and tetracycline (90.57%, 96/106) (Fig. 1D). Conversely, less than 15% of the isolates were resistant to ciprofloxacin (13.21%, 14/106), ceftiofur (9.43%, 10/106), gentamicin (5.66%, 6/106), imipenem (2.83%, 3/106), enrofloxacin (1.89%, 2/106), cefepime (0.94%, 1/106), and colistin (0.94%, 1/106). Regarding different antimicrobial classes, all *Salmonella* isolates recovered from pig slaughterhouses were resistant to macrolides (100%; erythromycin & tilmicosin) while a large proportion of the isolates were resistant to tetracyclines (97.17%; tetracycline & tigecycline & doxycycline), aminocyclitols (96.23%; spectinomycin), phenicols (79.25%; florfenicol), penicillins (78.30%; ampicillin & amoxicillin), and folate pathway antagonists (67.92%; sulfisoxazole & sulfamethoxazole) (Fig. 1E). In contrast, a low proportion of the isolates were resistant to fluoroquinolones (14.15%; ciprofloxacin & enrofloxacin), cephalosporins (9.43%; ceftiofur & cefepime), aminoglycosides (5.66%; gentamicin), carbapenems (2.83%; imipenem), and polymyxins (0.94%; colistin). Over 99.06% (105/106) of the isolates displayed phenotypes of multidrug resistance (resistant to more than 3 antimicrobial classes) and nearly half of them (42.86%, 42/105) were resistant to six of the eleven antimicrobial classes tested (Fig. 1F). Resistance to “macrolides plus tetracyclines plus aminocyclitols” was the most common multidrug resistant phenotypes, accounting for 94.29% (99/106) of the multidrug resistant isolates.

### Isolation and phenotypical characteristics of *Salmonella* bacteriophages

**Fig. 2:**
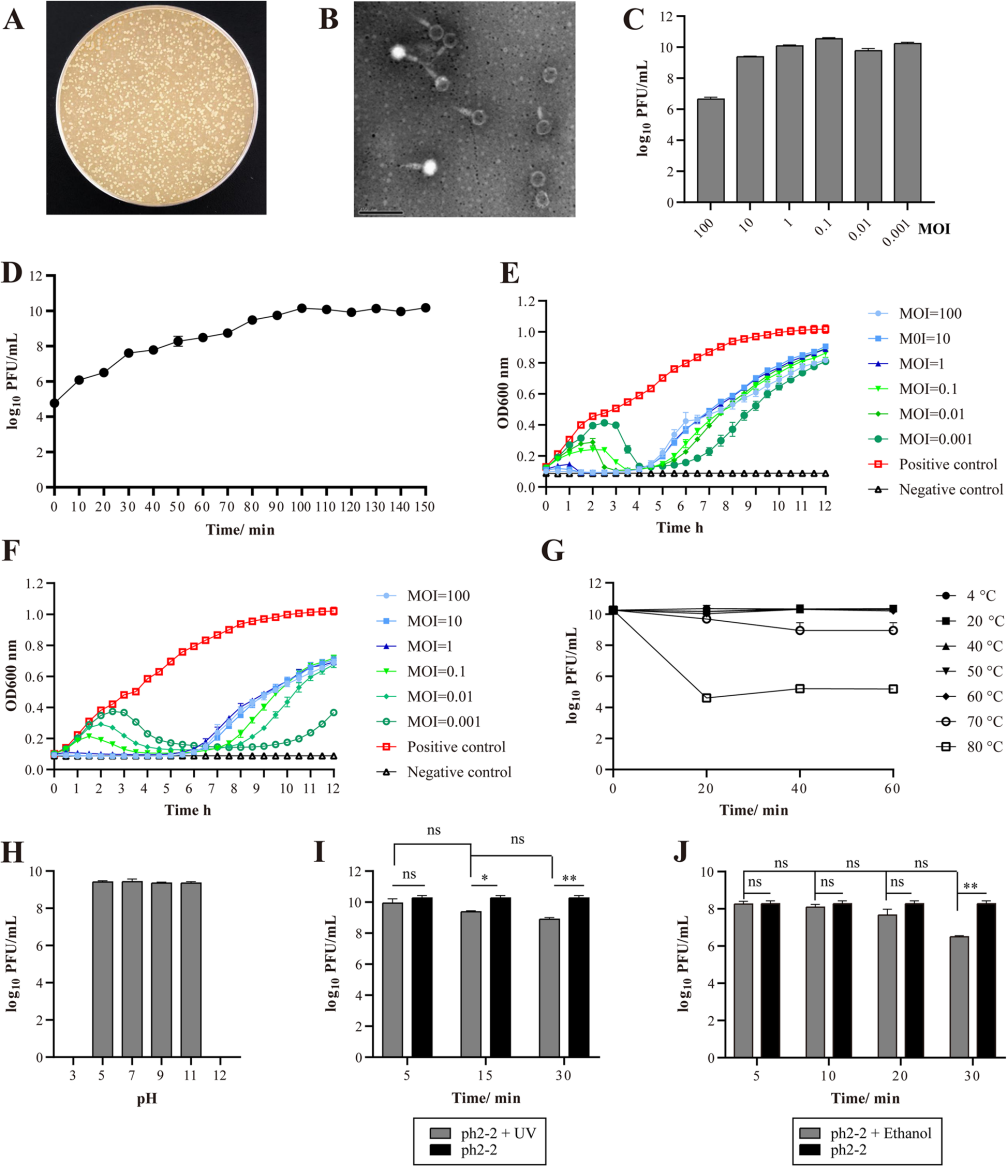
Phenotypical characteristics of *Salmonella* phage ph 2–2. **A** Plaques of phage ph 2–2 on *Salmonella Paratyphi* 201,007; **B** Transmission electron micrograph of phage ph 2–2; **C** A column chart showing the titers of phage ph 2–2 at different multiplicity of infection (MOI) values; **D** One-step growth curve of phage ph 2–2; **E** A line chart showing the effect of phage ph 2–2 killing *Salmonella Paratyphi* strain 201,107 at different MOI values; **F** A line chart showing the effect of phage ph 2–2 killing *Salmonella Typhimurium* 1344 at different MOI values; **G** A line chart showing the changes of ph 2–2 titers at different temperatures; **H** A column chart showing the changes of ph 2–2 titers at different pHs; **I** A column chart showing the changes of ph 2–2 titers exposed to UV for different times; **J** A column chart showing the changes of ph 2–2 titers exposed to 75% ethanol for different times. Data represents mean ± SD. The significance level was set at *P* < 0.05 (*) or *P* < 0.001 (**); ns: No significance

Using *Salmonella* isolates from slaughterhouses as indicator bacteria, we isolated 37 phages from pig anal swabs collected from different pig farms and slaughterhouses in Hubei Province. According to the plaque size formed by these phages, we selected one designated ph 2–2, which produced the largest and clearest plaques for further evaluation (Fig. 2A). Phage ph 2–2 was isolated using a *Salmonella Paratyphi* strain 201,107 as the indicator and a titer of 1.8 × 10^10^ PFU/ml was produced using the host bacterium. Electron microscopy showed that ph 2–2 had an icosahedrally symmetric head of approximately 70.00 nm in diameter and a long tail of ~ 110.00 nm in length (Fig. 2B). Based on these morphological characteristics and according to the latest International Committee on Taxonomy of Viruses (ICTV) classification, ph 2–2 was defined as a member of the *Siphoviridae* family.

We next tested different life cycle parameters of ph 2–2. Measurement of optimal multiplicity of infection (MOI) showed that ph 2–2 had the highest titer (3.0 × 10^10^ PFU/ml) in the host bacterium at MOI =0.1 (Fig. 2C). One-step-curve determination tests demonstrated that the life cycle of ph 2–2 consisted of an approximately 10-min eclipse period and a 90-min infection process; the average burst size was 476 phage particles per infected cell after 100 min at 37 °C (Fig. 2D). Test of bacteriophage lytic curve showed that ph 2–2 displayed good effects to lyse both the host bacterium *Salmonella Paratyphi* strain 201,107 and a *Salmonella Typhimurium* 1344 (Fig. 2E and F). Thermolability tests revealed ph 2–2 was stable from 4 ~ 60 °C, but it still exhibited lytic activities at 70 °C for 40 min or 80 °C for 20 min (Fig. 2G). pH sensitivity tests showed that ph 2–2 was stable from pH 5.0 to pH 11.0 (Fig. 2H). UV and ethanol exposure tests demonstrated that ph 2–2 still displayed good antibacterial effects after exposure to UV for 5 min (Fig. 2I), and/or treatment with 75% ethanol for 20 min (Fig. 2J).

Host range tests revealed that the phage was able to kill all the 106 *Salmonella* isolates from the slaughterhouses (Table 1). However, it displayed no capacity to lyse bacteria belonging to other species, including *Staphylococcus aureus*, *Escherichia coli*, *Enterococcus faecalis*, *Aeromonas hydrophila*, *Klebsiella pneumoniae*, *Bordetella bronchiseptica*, and *Streptococcus suis* (Table 1)*.*

**Table 1:** Host range of *Salmonella* phage ph 2–2

NO.	Strain	Bacterial species	EOP^**a**^	NO.	Strain	Bacterial species	EOP
1	200,701	*Salmonella* ^b^	+	58	201,140	*Salmonella Typhimurium*	+
2	200,901	*Salmonella*	+	59	201,141	*Salmonella Typhimurium*	+
3	200,902	*Salmonella*	+	60	201,142	*Salmonella*	+
4	200,903	*Salmonella*	+	61	210,401	*Salmonella Typhimurium*	+
5	200,904	*Salmonella*	+	62	210,402	*Salmonella Typhimurium*	+++
6	200,905	*Salmonella*	+	63	210,403	*Salmonella Typhimurium*	+++
7	200,906	*Salmonella Typhimurium*	+	64	210,404	*Salmonella Typhimurium*	+++
8	200,907	*Salmonella Typhimurium*	+	65	210,405	*Salmonella Typhimurium*	+++
9	200,908	*Salmonella Typhimurium*	+	66	210,407	*Salmonella Typhimurium*	++
10	200,909	*Salmonella*	+	67	210,408	*Salmonella Typhimurium*	++++
11	200,910	*Salmonella* Derby	+	68	210,409	*Salmonella Typhimurium*	++
12	201,001	*Salmonella*	+++	69	210,410	*Salmonella Typhimurium*	+++
13	201,002	*Salmonella Typhimurium*	+	70	210,411	*Salmonella Typhimurium*	++
14	201,003	*Salmonella Typhimurium*	+	71	210,412	*Salmonella Typhimurium*	+++
15	201,004	*Salmonella Typhimurium*	+	72	210,413	*Salmonella Typhimurium*	++
16	201,005	*Salmonella Typhimurium*	+	73	210,415	*Salmonella Typhimurium*	++++
17	201,006	*Salmonella Typhimurium*	++++	74	210,416	*Salmonella Paratyphi* A	+
18	201,007	*Salmonella Paratyphi* A	1 ^c^	75	210,417	*Salmonella Typhimurium*	+
19	201,101	*Salmonella Typhimurium*	++	76	210,418	*Salmonella Typhimurium*	++++
20	201,102	*Salmonella Paratyphi* A	+	77	210,419	*Salmonella Typhimurium*	++++
21	201,103	*Salmonella Typhimurium*	+++	78	210,420	*Salmonella Typhimurium*	++
22	201,104	*Salmonella Typhimurium*	++++	79	210,421	*Salmonella Typhimurium*	++++
23	201,105	*Salmonella Typhimurium*	+++	80	210,422	*Salmonella Typhimurium*	++++
24	201,106	*Salmonella*	+++	81	210,424	*Salmonella Typhimurium*	++++
25	201,107	*Salmonella Typhimurium*	++++	82	210,425	*Salmonella Typhimurium*	++
26	201,108	*Salmonella Typhimurium*	+	83	210,426	*Salmonella Typhimurium*	++++
27	201,109	*Salmonella Paratyphi* A	+++	84	210,427	*Salmonella Typhimurium*	++++
28	201,110	*Salmonella* Infantis	+	85	210,429	*Salmonella Typhimurium*	+++
29	201,111	*Salmonella Typhimurium*	++	86	210,430	*Salmonella Typhimurium*	+++
30	201,112	*Salmonella Typhimurium*	+	87	210,431	*Salmonella Typhimurium*	+++
31	201,113	*Salmonella Typhimurium*	+++	88	210,433	*Salmonella Typhimurium*	+++
32	201,114	*Salmonella* Infantis	+	89	210,434	*Salmonella Typhimurium*	++++
33	201,115	*Salmonella* Infantis	+	90	210,435	*Salmonella Typhimurium*	+++
34	201,116	*Salmonella*	+	91	210,436	*Salmonella Typhimurium*	++++
35	201,117	*Salmonella Typhimurium*	++	92	210,437	*Salmonella Typhimurium*	+++
36	201,118	*Salmonella*	+	93	210,438	*Salmonella Typhimurium*	++
37	201,119	*Salmonella Typhimurium*	+	94	210,439	*Salmonella Paratyphi* A	+
38	201,120	*Salmonella*	+	95	210,440	*Salmonella Paratyphi* A	+
39	201,121	*Salmonella Typhimurium*	++	96	210,501	*Salmonella Typhimurium*	+
40	201,122	*Salmonella Paratyphi* A	+	97	210,502	*Salmonella Typhimurium*	+
41	201,123	*Salmonella Typhimurium*	+++	98	210,503	*Salmonella Typhimurium*	+
42	201,124	*Salmonella*	+++	99	210,504	*Salmonella Typhimurium*	+
43	201,125	*Salmonella*	+	100	210,505	*Salmonella Typhimurium*	+
44	201,126	*Salmonella Paratyphi* A	+	101	210,506	*Salmonella Typhimurium*	+
45	201,127	*Salmonella Typhimurium*	+++	102	210,507	*Salmonella Typhimurium*	++
46	201,128	*Salmonella Typhimurium*	+	103	210,508	*Salmonella Typhimurium*	+
47	201,129	*Salmonella Typhimurium*	++	104	210,701	*Salmonella Typhimurium*	++++
48	201,130	*Salmonella Paratyphi* A	+	105	210,702	*Salmonella Typhimurium*	++
49	201,131	*Salmonella Typhimurium*	+	106	210,703	*Salmonella Typhimurium*	++
50	201,132	*Salmonella Typhimurium*	+	107	SA25	*Staphylococcus aureus*	–
51	201,133	*Salmonella Typhimurium*	+	108	E02	*Escherichia coli*	–
52	201,134	*Salmonella*	+	109	EF-3	*Enterococcus faecalis*	–
53	201,135	*Salmonella Typhimurium*	+	110	AH01	*Aeromonas hydrophila*	–
54	201,136	*Salmonella Typhimurium*	+	111	KP6	*Klebsiella pneumoniae*	–
55	201,137	*Salmonella Typhimurium*	+	112	HN05	*Pasteurella multocida*	–
56	201,138	*Salmonella Typhimurium*	+++	113	Bb-5	*Bordetella bronchiseptica*	–
57	201,139	*Salmonella*	+	114	SS-1	*Streptococcus suis*	–

^a^*EOP* efficiency of plating, which was determined by calculating the ratio of plaque-forming units (PFUs) of each phage-susceptible strain to the PFUs of indicator strain (*Salmonella Paratyphi* 210,007); “++++”: EOP > 1; “+++”: 1 ≥ EOP > 0.1; “++”: 0.1 ≥ EOP > 0.001; “+”: EOP ≤ 0.001; “-”: EOP = 0

^b^If a serovar is not determined then the strain is marked as *Salmonella* only

^c^The EOP of the indicator bacterium of ph 2–2 is marked as 1

### Genomic characteristics of a lytic *Salmonella* bacteriophage

**Fig. 3:**
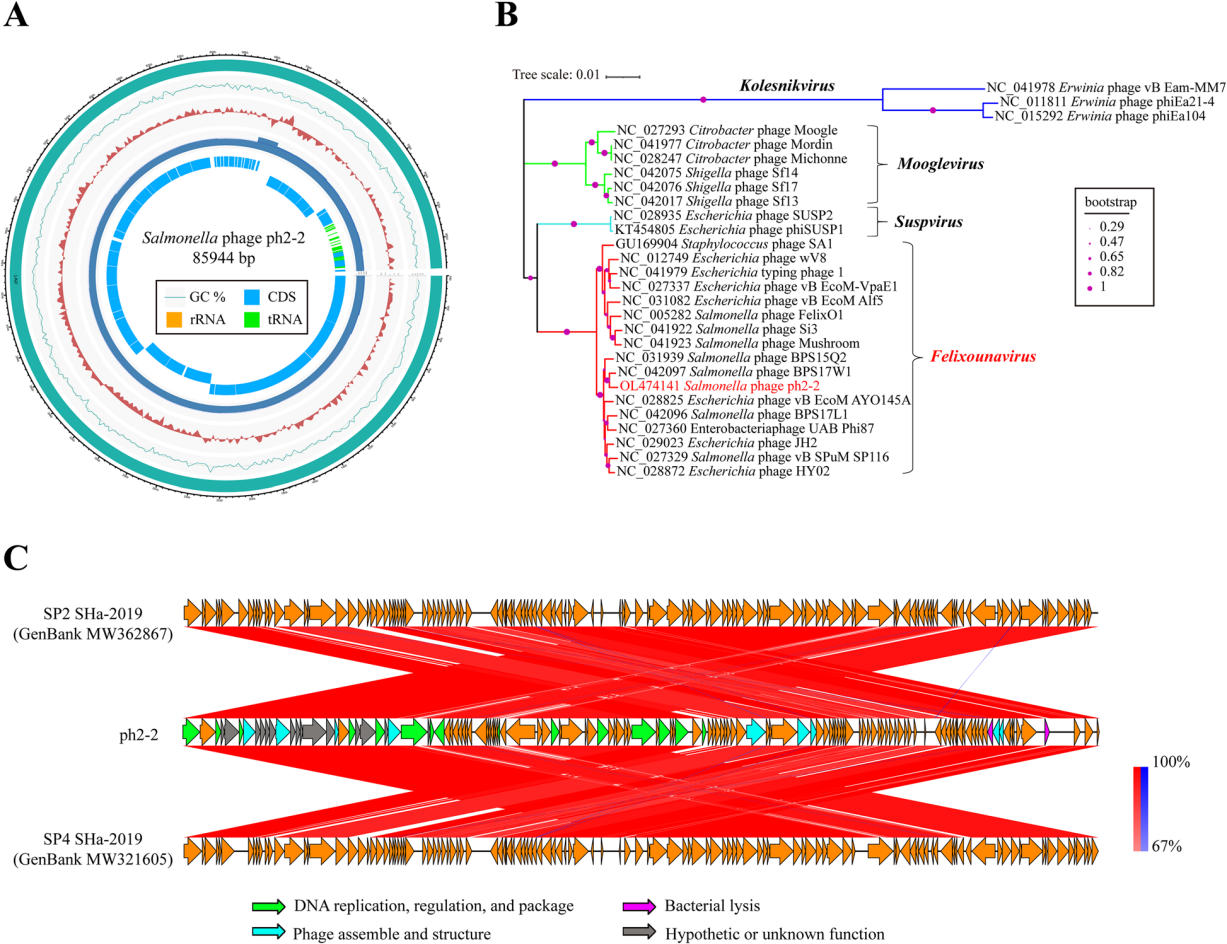
Genomic characteristics of *Salmonella* phage ph 2–2. **A** A circle map showing the complete genome sequence of phage ph 2–2; circles from inside to outside represent the numbers of coding sequences (CDS) and tRNAs (circle 1), depth of illumine sequencing (circle 2), GC skew (circle 3), G + C content (circle 4), and the genome circle (circle 5); **B** Phylogenetic relationships of bacterial phages belonging to the *Myoviridae* family; the tree was generated based on the nucleotide sequences of the large subunit of phage terminases; The evolutionary distances were computed using the Maximum Composite Likelihood method and are in the units of the number of base substitutions per site; The optimal tree with the sum of branch length = 0.31527374 is shown; The percentage of replicate trees in which the associated taxa clustered together in the bootstrap test (1000 replicates) are shown next to the branches; There were a total of 1605 positions in the final dataset. Evolutionary analyses were conducted in MEGA X; (**C**) A co-linearity comparison diagram of the genomic organization at the nucleotide level between *Salmonella* phages ph 2–2, SP2 SHa-2019 (GenBank accession number: MW362867) and SP4 SHa-2019 (GenBank accession number: MW321605); The figure was generated via Easyfig v.2.0. The color code refers to the BLASTn identity of those regions between genomes. Arrows represent putative CDSs encoded by different genomes

Whole genome sequencing demonstrated that ph 2–2 possessed a double-strand genomic DNA of approximately 85,944 bp in length with a G + C content of 38.81% (Fig. 3A). The genome of ph 2–2 encoded 128 putative proteins involved in phage structure and assembly, DNA replication and regulation modules, lysis function, and/or unknown function (Table S1 in supplementary file). Phylogenetic analysis based on the nucleotide sequences of the large subunit of phage terminase showed that ph 2–2 was a member of the *Felixounavirus* genus of the *Myoviridae* family (Fig. 3B). Sequence alignments revealed that the genome sequence of ph 2–2 was highly homologous to those of *Salmonella* phage SP2 SHa-2019 (GenBank accession number: MW362867) and *Salmonella* phage SP4 SHa-2019 (GenBank accession number: MW321605) (Fig. 3C). The average nucleotide identity (ANI) between the genomes of ph 2–2 and SP2 SHa-2019 was 95.38% (calculated by ANI, http://enve-omics.ce.gatech.edu/ani/), and 95.38% between the genomes of ph 2–2 and SP4 SHa-2019. However, the genome of ph 2–2 encoded two putative lysozymes, while both the genomes of SP2 SHa-2019 and SP4 SHa-2019 encoded one lysozyme (Fig. 3C).

### Application of a lytic bacteriophage to control *Salmonella* infections in mouse models

**Fig. 4:**
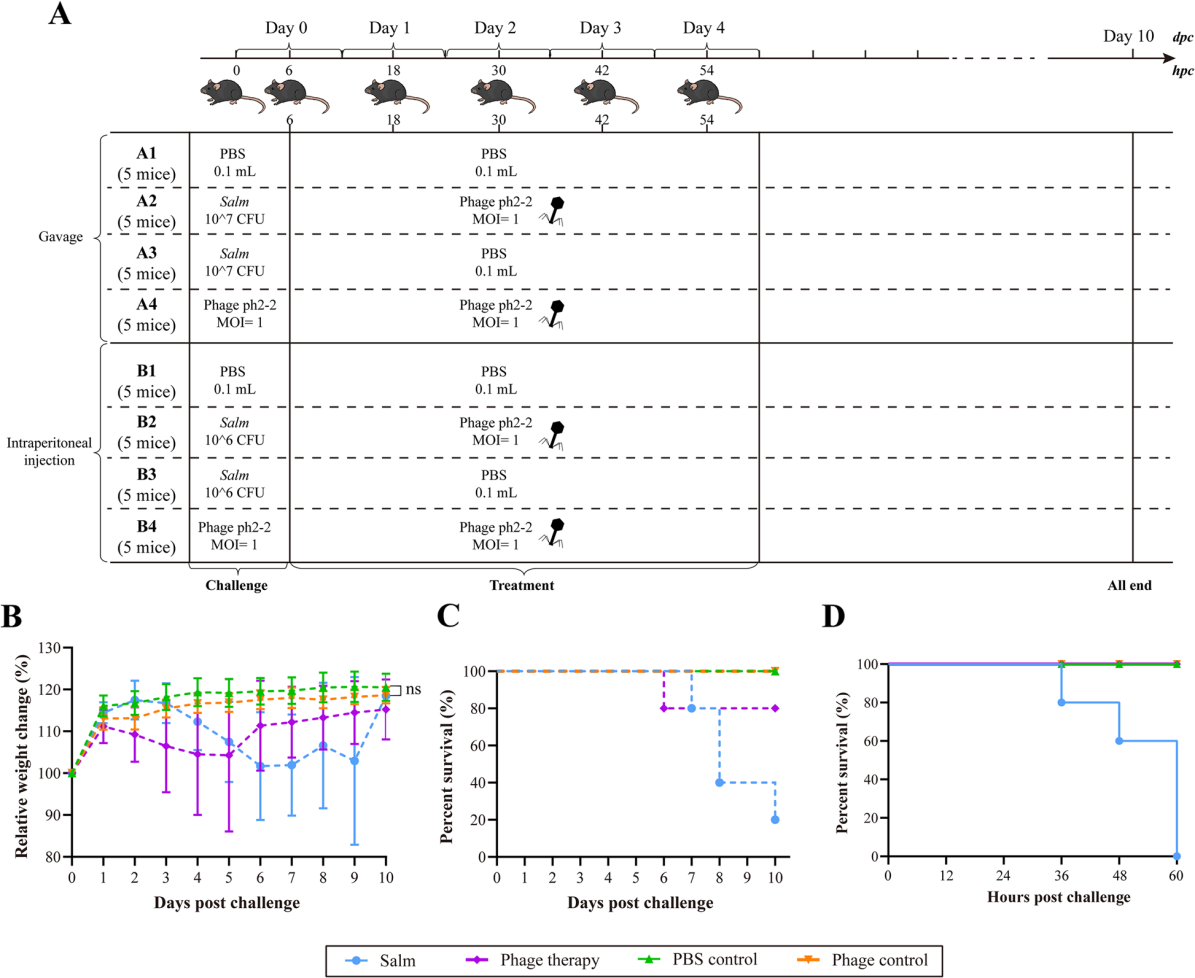
Experimental scheme for the evaluation of ph 2–2 treatment efficacy in mice infected with *Salmonella Typhimurium* 1344. **A** Study design of the animal tests; dpc: days post challenge; hpc: hours post challenge; **B** A line chart showing changes of body weight of mice challenged with *Salmonella Typhimurium* 1344 by gavage and received a treatment of PBS by gavage (blue line); mice challenged with *Salmonella Typhimurium* 1344 by gavage and received a treatment of ph 2–2 by gavage (purple line); mice received an administration of PBS (green line) and/or ph 2–2 by gavage (orange line); Data represents mean ± SD. ns: No significance; **C** Mortality of mice challenged with *Salmonella Typhimurium* 1344 by gavage and received a treatment of PBS by gavage (blue line); mice challenged with *Salmonella Typhimurium* 1344 by gavage and received a treatment of ph 2–2 by gavage (purple line); mice received an administration of PBS (green line) and/or ph 2–2 by gavage (orange line); **D** Mortality of mice challenged with *Salmonella Typhimurium* 1344 through intraperitoneal injection and received a treatment of PBS through intraperitoneal injection (blue line); mice challenged with *Salmonella Typhimurium* 1344 through intraperitoneal injection and received a treatment of ph 2–2 through intraperitoneal injection (purple line); mice received an administration of PBS (green line) and/or ph 2–2 through intraperitoneal injection (orange line)

To further investigate the activity of ph 2–2 on the control of *Salmonella* infection, 4–6-week-old C57BL/6 J mice were challenged with *Salmonella Typhimurium* 1344 through gavage (~ 10^7^ CFU per mouse) and intraperitoneal routine (~ 10^6^ CFU per mouse), and then received a treatment of either ph 2–2 (10^7^ PFU per mouse, MOI = 1) or PBS (Fig. 4A). In parallel, mice were also treated with ph 2–2 or PBS only by gavage or through the intraperitoneal routine. The results revealed a good safety of ph 2–2 to the mice, as the administration of the phage, either by gavage or intraperitoneal routine, did not affect the growth condition and/or lead to the death of the mice (Fig. 4B, C, and D). Moreover, ph 2–2 therapy, either by gavage or through intraperitoneal administration, dramatically lessened body-decrease caused by *Salmonella* (Fig. 4B), and reduced the mortality by *Salmonella* (Fig. 4C and D). Treatment of ph 2–2 by gavage saved 75% (3/4) of the mice from *Salmonella* infection by gavage (Fig. 4C), while intraperitoneal administration of ph 2–2 saved 100% (5/5) of the mice from *Salmonella* infection by intraperitoneal challenge (Fig. 4D).

## Discussion

In this study, we investigated the prevalence of *Salmonella*, which is a very important foodborne and zoonotic pathogen, in eleven slaughterhouses in seven cities in Hubei Province. Our results revealed that *Salmonella* isolates could be recovered from different types of samples collected from these pig slaughterhouses, indicating that contamination of *Salmonella* represents a problem in the pig slaughtering node of the pork supply chain. Considering pork is the primary meat for most of the people in China [15], the prevalence of *Salmonella* in pig slaughterhouses should receive more attention, and actions should be taken to decrease the contamination of *Salmonella*. This is particularly important as *Salmonella* is responsible for 37.3% of foodborne bacterial diseases in China [16]. Our data of bacterial isolation also revealed that many *Salmonella* isolates were recovered from the anal swabs of pigs, suggesting that the pig farms might be an important origin for *Salmonella* contamination in slaughterhouses. In the next step, we intend to investigate the contamination of *Salmonella* in the upstream pig farms of those *Salmonella*-recovered pigs. Our determination of serovars showed that most *Salmonella* isolates recovered from slaughterhouses were *Salmonella Typhimurium*. These results are in agreement with those from other studies performed in both China and outside China [17–19]. It should be noted that *Salmonella Typhimurium* has been recognized to be responsible for the majority of *Salmonella* infection cases worldwide [5]. Therefore, the contamination of this serovar poses a big threat to public health. This study also recovered nine *Salmonella Paratyphi* isolates from both pig anal swabs (*n* = 6) and environmental samples (*n* = 3) from slaughterhouses (Fig. 1C). Among different *Salmonella Paratyphi* members, *Salmonella Paratyphi* A strains are host-restricted pathogens whose reservoir is humans [20]; while other *Salmonella Paratyphi* sub-serovars such as B or C strains have been widely recovered from non-human hosts [21, 22]. In the next step, we intend to study the sub-serovars, virulence and genomics of these nine *Salmonella Paratyphi* isolates.

Administration of antibiotics is still an effective option for the treatment of bacterial infections in both human and veterinary medicine [23]. However, the emergence and dissemination of antibiotic resistant bacteria may lead to antibiotic-based therapy failure in clinical activity and therefore have raised a global public health concern in recently years [7]. Since food animals are considered as key reservoirs of antibiotic-resistant bacteria [24], we determined the AMR phenotypes of *Salmonella* isolates recovered in this study, and our AST results indicated a high rate of antimicrobial resistance of these isolates. Many isolates displayed resistance phenotypes to macrolides, tetracyclines, aminocyclitols, phenicol, penicillin, and folate pathway antagonists. While most of these antibiotics are not used in slaughterhouses, they are frequently used in pig farms in China [25–27]. The extensive use of these antibiotics in farms may induce the resistance phenotypes in *Salmonella*, and these drug-resistant *Salmonella* are finally recovered from the swabs of pigs shipped to the slaughter houses. It should be noted that a large proportion of isolates (80.19%, 85/106) were found to be tigecycline-resistant. This might be because currently only a EUCAST breakpoint for tigecycline is available, and this value is very low (Resistant enterobacteria are interpreted as those with a MIC value over 0.5 μg/ml) [28]. Most of the tigecycline-resistant *Salmonella* recovered in this study possessed MIC values of 1 μg/ml (39 isolates) or 2 μg/ml (31 isolates). While as a last-resort antibiotic for treating infections caused by gram-negative bacteria, tigecycline has never been approved to be used in agriculture in China, but tetracycline-resistant bacteria displaying tigecycline-resistance at low level have been documented [29–31]. Therefore, the phenotypes of tigecycline-resistance determined in these isolates might be associated with their tetracycline-resistance. As another kind of last-resort antibiotic, imipenem has also never been approved to be used in livestock in China, several imipenem-resistant isolates were still recovered. The recovery of these isolates might due to contaminated in-house environment, as a recent study have found a high detection rate (26.8–31.4%) of *bla*_NDM_ (which confers resistance to carbapenems) in environmental samples except air after standard cleaning and disinfection during the vacancy period in a Chinese poultry farm [32]. While it still lacks of direct evidence, similar conditions might also occur in pig farms. In addition, we also recovered several colistin-resistant *Salmonella* isolates. Although colistin has been banned for use in agriculture in China in 2017 [33], colistin-resistant bacteria or genes (e.g., the *mcr* family) may persist in livestock in China [34–36]. In the next step, we intend to analyze the molecular mechanisms of resistance to these last-resort antibiotics in the *Salmonella* isolates recovered in this study.

As the natural predators of bacteria, phages are recognized as promising therapeutics for bacterial infections since their discovery [12], and they indeed have achieved a great success in saving lives from infections caused by MDR-pathogens [13]. While there is still a long way to go, many laboratory studies have tested the potential use of phages or their related products in fighting against bacteria, and those studies have also demonstrated good results [37–39]. Therefore, we also isolated and screened lytic phages using the *Salmonella* isolates as indictor bacteria and evaluated their use in combating the drug resistant *Salmonella* recovered in this study. According to the results of a series of laboratory tests, a lytic phage ph 2–2 demonstrated a good potential. This phage was stable and displayed good capacity of killing drug resistant *Salmonella* in different adverse conditions (high or low temperatures, high or low pHs, UV exposure, 75% Ethanol exposure). In particular, the burst size, thermolability, and pH stability of ph 2–2 are better than those of the three *Salmonella* phages we tested previously [40]. A good stability of a phage in different adverse conditions increases its potential use in fighting against pathogenic bacteria [37, 38, 40, 41]. In addition to good stability, a potential phage candidate should also have a wide host range [37, 38, 40]. Our test revealed that although ph 2–2 was isolated using a *Salmonella Paratyphi* strain, it displayed good capacity to kill *Salmonella* isolates belonging to other serovars, including *Salmonella Typhimurium*. Considering *Salmonella Typhimurium* is the causative agent of the majority of *Salmonella* infection cases worldwide [5], we therefore investigated the effect of ph 2–2 on treating *S. typhimurium* infections in mouse models in different administration routines. Our results showed that ph 2–2 was safe to mice and could save experimental mice from lethal infections caused by *Salmonella Typhimurium*. These findings indicate that ph 2–2 might be a good candidate to combat drug-resistant *Salmonella* in vivo and in vitro.

## Conclusions

The data presented herein demonstrated that *Salmonella* contamination remains a problem in some pig slaughter houses in China and *Salmonella* isolates recovered from pigs or environmental samples of slaughter houses displayed a high rate of antimicrobial resistance. In addition, we also showed a broad-spectrum lytic *Myoviridae* phage ph 2–2 displayed good capacity to kill drug resistant *Salmonella* in vivo and in vitro*.* It might represent a good candidate for the development of anti-*Salmonella* agents.

## Methods

### Sample collection, bacterial isolation, purification, and serotyping

Between July 2020 and July 2021, a total of 1289 samples including anal swabs of pigs (862/1289), environmental swabs (204/1289), carcass surface swabs (36/1289) and environmental agar plates (187/1289; *Salmonella Shigella* [SS] Agar plates were left in the open for at least 3 hours in different spaces along the pig treatment direction in the slaughterhouses) were collected from eleven slaughterhouses in seven cities in Hubei Province in China (Fig. 1A). Swabs were stored in Buffered Peptone Water (BPW). All samples were shipped to laboratory on ice and were treated immediately after collection. *Salmonella* was isolated as described previously [42]. Briefly, swabs were streaked on SS agars and were incubated at 37 °C for 24 h. Environmental agar plates were put into a 37 °C chamber directly. Presumptive colonies were selected and *Salmonella* was confirmed by gram-staining, biochemical tests, 16S rRNA sequencing, as well as PCR detection of the *invA* gene as described previously [43]. A previously reported multiplex PCR serotyping method was applied to determine the serovars of *Salmonella* isolates recovered in this study [44]. The determined serovars were finally confirmed through the Kauffmann–White classification method [45]. *Salmonella* antisera were purchased from Ningbo Tianrun Bio-pharmaceutical Co., LTD (Ningbo, China).

### Antimicrobial susceptibility testing

Antimicrobial susceptibility testing was performed using broth microdilution method following the protocol published by Clinical & Laboratory Standards Institute (CLSI) [46]. The minimum inhibitory concentration (MIC) values of 18 types of antibiotics belonging to aminocyclitols (spectinomycin; MedChemExpress [MCE], Monmouth Junction, US), aminoglycosides (gentamicin; MCE), carbapenems (imipenem; MCE), cephalosporins (ceftiofur, cefepime; MCE), fluoroquinolones (ciprofloxacin, enrofloxacin; MCE), folate pathway antagonists (sulfisoxazole, sulfamethoxazole; MCE), macrolides (erythromycin, tilmicosin; MCE), penicillins (ampicillin, amoxicillin; MCE), phenicols (florfenicol; MCE), polymyxins (colistin; MCE), and tetracyclines (tetracycline, tigecycline, doxycycline; MCE). Results were interpreted using CLSI breakpoints (CLSI M100: amoxicillin [Resistant (R): ≥ 32 μg/ml, Intermediate (I): 16 μg/ml, Susceptible (S): ≤ 8 μg/ml]; ampicillin [R: ≥ 32 μg/ml, I: 16 μg/ml, S: ≤ 8 μg/ml]; cefepime [R: ≥ 16 μg/ml, S: ≤ 2 μg/ml]; ciprofloxacin [R: ≥ 1 μg/ml, I: 0.12–0.5 μg/ml, S: ≤ 0.06 μg/ml]; colistin [R: ≥ 4 μg/ml, I: 2 μg/ml]; doxycycline [R: ≥ 16 μg/ml, I: 8 μg/ml, S: ≤ 4 μg/ml]; gentamicin [R: ≥ 16 μg/ml, I: 8 μg/ml, S: ≤ 4 μg/ml]; sulfamethoxazole [R: ≥ 76 μg/ml, S: ≤ 8 μg/ml]; sulfisoxazole [R: ≥ 512 μg/ml, S: ≤ 256 μg/ml]; imipenem [R: ≥ 4 μg/ml, I: 2 μg/ml, S: ≤ 1 μg/ml]; tetracycline [R: ≥ 16 μg/ml, I: 8 μg/ml, S: ≤ 4 μg/ml]; CLSI M31-A3: ceftiofur [R: ≥ 8 μg/ml, I: 4 μg/ml, S: ≤ 2 μg/ml]; enrofloxacin [R: ≥ 4 μg/ml, I: 1–2 μg/ml, S: ≤ 0.5 μg/ml]; florfenicol [R: ≥ 16 μg/ml, I: 8 μg/ml, S: ≤ 4 μg/ml]) [46, 47], or EUCAST breakpoints (tigecycline [R: > 0.5 μg/ml, S: ≤ 0.5 μg/ml]) [28], or by reference published articles (erythromycin [R: ≥ 8 μg/ml]; spectinomycin [R: ≥ 32 μg/ml]; tilmicosin [R: ≥ 32 μg/ml]) [48]. For each type of the antibiotics, the MIC value was tested three times separately. *E. coli* ATCC 25922 was used as quality control.

### Bacteriophage isolation and purification

Bacteriophages were isolated from 83 anal swabs of pigs collected from slaughterhouses and farms in Hubei Province through a previously described double-layer agar method [37, 38], with several minor modifications. *Salmonella* isolates recovered in this study were used as indicator bacteria. Briefly, anal swabs were washed thoroughly using PBS. The mixtures were centrifuged at 7000 rpm for 10 min, followed by a filtration through a 0.22-μm pore size membrane. After that, the filtrates, the bacterial culture of *Salmonella* at mid-log phase, and fresh Luria-Bertani (LB) broth (Thermo Fisher Scientific, Waltham, MA) were mixed at a volume ratio of 1: 1: 2. The mixture was shaken at 220 rpm, 37 °C for 2.5–3.5 h. The above-cocultures were rested at 4 °C for 2 h, followed by another centrifugation at 4 °C, 7000 rpm for 10 min. The supernatants were filtered again through a 0.22-μm pore size membrane. Thereafter, the filtrate was mixed with the indicator bacterium at a volume ratio of 1: 3, and was poured into 8 ml of molten soft LB agar (LB broth + 1.5% *w*/*v* agar [final concentration]). Finally, the mixture was poured onto a prepared Tryptic Soy Agar (TSA; Sigma-Aldrich, St. Louis, US) and incubated overnight at 37 °C to numerate the plaques.

After the plaques were numerated, a single plaque was picked and resuspended using a SM buffer [5.8 g of NaCl, 2.0 g of MgSO_4_·7H_2_O, 50 mL of Tris-HCl (pH 7.4), 5.0 mL of 2% gelatin] [49]. After centrifugation at 12,000 rpm for 30 s, the supernatant of the phage-containing SM buffer was filtered through a 0.22-μm pore size membrane. Next, the phage preparations were given serial 10-fold dilutions with sterile SM buffer. Phage isolation by above-mentioned double-layer agar method was repeated four more times, and the phage suspensions were stored at 4 °C. Finally, the phages were purified by CsCl gradient ultra-centrifugation, as described previously [41].

### Phenotypical characterization

To determine the morphology of ph 2–2, samples were prepared according to the protocol described previously [41], and were observed under a 100-kV transmission electron microscope (HITACHI H-7650, Tokyo, Japan). To measure the optimal MOI value, ph 2–2 at different MOI values (0.001, 0.01, 0.1, 1.0, 10.0) were incubated with the indicator bacterium *Salmonella Paratyphi* strain 201,107 at mid-log phase (2.94 × 10^7^ CFU) in LB broth at 37 °C, 180 rpm for 3 h, and the optimal MOI value was determined through the above-mentioned double-layer agar method. For the measurement of the one-step growth curve, ph 2–2 at optimal MOI value was co-cultured with *Salmonella Paratyphi* 201,107 at mid-log phase. After that, phage titers were measured once every 10 min for 150 min. The experiment was repeated three times, and the burst size was calculated as the ratio between the number of phages before and after the burst [37, 38]. The thermolability of ph 2–2 was tested by measuring the titers of the phage following treatments of the phage particles (in SM buffer) at different temperatures (4 °C, 20 °C, 40 °C, 50 °C, 60 °C, 70 °C, and/or 80 °C) from different times (20 min, 40 min, and 60 min); while the pH sensitivity was tested by measuring the titers of the phage following incubations of the phage particles (in SM buffer) at 37 °C for 1 h under different pH levels (3, 4, 5, 6, 7, 8, 9, 10, 11, and 12). To test the UV sensitivity, phage particles were treated under UV (20 W) for different times (0, 5, 15, 30 min), and were then treated at dark atmosphere for another 30 min before measuring the titers. For the determination of ethanol sensitivity, phage particles were treated using 75% ethanol and the titers were measured every 10 min post the treatment. In the above tests of thermolability, pH sensitivity, UV sensitivity, and ethanol sensitivity, samples were titered by the double-layer agar plate method [37], and each assay was performed in triplicate.

The host range of ph 2–2 was determined by spot tests, as described previously [50]. All 106 *Salmonella* isolates recovered in this study as well as our laboratory stored eight strains belonging to the other bacterial species (*Staphylococcus aureus*, *Escherichia coli*, *Enterococcus faecalis*, *Aeromonas hydrophila*, *Klebsiella pneumoniae*, *Bordetella bronchiseptica*, and *Streptococcus suis*) were used (Table 1). Each of the bacterial strains at mid-log phase were mixed with the above-mentioned molten soft LB agar at a volume ratio of 1:3, which was then poured onto a prepared Tryptic Soy Agar. After each overlay solidified, 4 μL of the phage lysate (1 × 10^10^ PFU/mL) was spotted onto the bacterial overlays, dried, and then incubated at 37 °C for 8 h. The same volume of sterile phage buffer was also spotted onto the bacterial overlays and incubated under the same conditions as the controls. Lytic specificity was defined based on the formation of bacteriophage plaques. The spot tests were repeated three times to confirm the results. The efficiency of plating (EOP) value was calculated as previously described [37], which was determined by calculating the ratio of plaque-forming units (PFUs) of each phage-susceptible strain to the PFUs of indicator strain (*Salmonella Paratyphi* 210,007). This experiment was also repeated three times.

### Whole genome sequencing, data availability, and bioinformatic analysis

Genomic DNA was extracted using the phenol-chloroform method, as described previously [37]. DNA quality and quantity was analyzed by electrophoresis on a 1% agarose gel as well as using a Qubit 2.0 (Thermo Scientific, Waltham, USA). Afterwards, 300–400 bp sequencing libraries were prepared using a commercial Agencourt AMPure XP medium kit, and were sequenced on a BGI MGISEQ-2000 platform (BGI, Shenzhen, China) according to the manufacturer’s protocol. A total of 643,053,476 bp raw reads (sequence coverage: 7482 ×) were yielded. Thereafter, raw reads with low quality were filtered and eliminated by SOAPnuke (version 1.5.0) software [51] according to the following criteria: reads with a certain proportion of low-quality bases (40% as the default, parameter setting at 20 bp), and/or with a certain proportion of Ns (10% as the default, parameter setting at 1 bp) were removed. Adapter contamination (15 bp overlap between the adapter and reads as the default, parameter setting at 15 bp) and duplication contamination were also removed. Through this step, approximately 643,053,476 bp clean reads (Q20% = 100%) were produced. These high-quality reads were de novo assembled using Unicycler package (version 0.4.8) [52]. Finally, an 85,944 bp (*N*_50_: 85,944 bp) genome sequence was obtained. Genome annotation was performed using RAST sever [53]. The complete genome sequence of ph 2–2 and its annotations have been deposited into GenBank, the accession number is OL474141. To clarify the taxonomical characteristics of ph 2–2, the nucleotide sequences of the large subunit of terminase were extracted from the whole genome sequences of different phages downloaded from NCBI (accession numbers are given in Fig. 3B). A phylogenetic tree generated based on the sequences of the large subunit of phage terminase was conducted in MEGA X [54] with a bootstrap value of 1000. Sequence alignment was performed and visualized using EasyFig v. 2.2.2 [55]. Average nucleotide identities between two genome sequences were calculated using an ANI calculator (http://enve-omics.ce.gatech.edu/ani/).

### Animal tests and ethic statement

All experiments were carried out in accordance with relevant guidelines and regulations, and the study was carried out in compliance with the ARRIVE guidelines. Mouse experiments were performed at the Laboratory Animal Center of Huazhong Agricultural University (Wuhan, China) with the approval from the Institutional Ethics Committees (IECs) of the University (approval number: HZAUMO-2021-0143). Laboratory animals were treated following the Regulations on the Administration of Laboratory Animals in Hubei Province [2005]. Study design is shown in Fig. 4A. Briefly, forty 4–6-week-old C57BL/6 J mice were divided into eight groups (A1 ~ A4; B1 ~ B4) and each group contained 5 mice. Mice in groups A2 and A3 were challenged with *Salmonella Typhimurium* 1344 (10^7^ CFU per mouse) by gavage while those in groups B2 and B3 were challenged with *Salmonella Typhimurium* 1344 (10^6^ CFU per mouse) through intraperitoneal routine. At 6-, 18-, 30-, 42-, and 54-hours post challenge (hpc), bacterial-infected mice in groups A2, A3, B2, and B3 received a treatment of phage ph 2–2 (10^7^ PFU per mouse by gavage), PBS (0.1 ml per mouse by gavage), phage ph 2–2 (10^7^ PFU per mouse through intraperitoneal injection), and PBS (0.1 ml per mouse through intraperitoneal injection), respectively. At the same time points, mice in groups A1, B1, A4, and B4 were administrated with PBS (0.1 ml per mouse) by gavage, ph 2–2 (10^7^ PFU per mouse) by gavage, PBS (0.1 ml per mouse) through intraperitoneal injection, phage ph 2–2 (10^7^ PFU per mouse) through intraperitoneal injection, respectively. Body weights and mortality of the experimental mice in each group were recorded.

### Statistical analysis

Statistical analysis was performed through the “Two-way ANOVA” strategy in GraphPad Prism8.0 (GraphPad Software, San Diego, CA). Data represents mean ± SD. The significance level was set at *P* < 0.05 (*).

## Supplementary Information

**Additional file 1: Table S1.** Putative proteins encoded by the genome sequence of *Salmonella* phage ph 2–2.

## Data Availability

The complete genome sequence of ph 2–2 and its annotations have been deposited into GenBank, the accession number is OL474141.

## Abbreviations

AMR: Antimicrobial resistance
AST: Antimicrobial susceptibility testing
CFU: Colony-forming unit
LB: Luria-Bertani
MOI: Multiplicity of infection
PFU: Plaque-forming unit
