# Supplementary material for: Identification of a broad-spectrum lytic Myoviridae bacteriophage using multidrug resistant Salmonella isolates from pig slaughterhouses as the indicator and its application in combating Salmonella infections
Source: BMC Vet Res. 2022 Jul 12;18:270. doi: 10.1186/s12917-022-03372-8 (PMC9277904; doi:10.1186/s12917-022-03372-8)
Supplement: Supplementary file 1 — Additional file 1: Table S1. Putative proteins encoded by the genome sequence of Salmonella phage ph 2–2. [file 12917_2022_3372_MOESM1_ESM.pdf]

Table S1 Putative proteins encoded by the genome sequence of *Salmonella* phage ph2-2.

ORF ID	Type	Start	Stop	Strand	Putative function
ORF1	Protein encoding gene	1	1602	+	Phage terminase, large subunit
ORF2	Protein encoding gene	1619	3085	+	Phage protein
ORF3	Protein encoding gene	3085	3585	+	Phage protein
ORF4	Protein encoding gene	3585	3917	+	Phage protein
ORF5	Protein encoding gene	3929	5275	+	Phage protein
ORF6	Protein encoding gene	5287	5664	+	Phage protein
ORF7	Protein encoding gene	5698	6804	+	Phage protein
ORF8	Protein encoding gene	6825	7274	+	Phage protein
ORF9	Protein encoding gene	7274	7756	+	Phage protein
ORF10	Protein encoding gene	7753	8154	+	Phage protein
ORF11	Protein encoding gene	8129	8728	+	Phage protein
ORF12	Protein encoding gene	8729	10081	+	Phage protein
ORF13	Protein encoding gene	10097	10543	+	Phage protein
ORF14	Protein encoding gene	10617	11015	+	Phage protein
ORF15	Protein encoding gene	11018	11257	+	Phage protein
ORF16	Protein encoding gene	11257	13467	+	Phage tail tape measure
ORF17	Protein encoding gene	13467	14264	+	Phage protein
ORF18	Protein encoding gene	14264	14605	+	Phage protein
ORF19	Protein encoding gene	14605	15582	+	Phage protein
ORF20	Protein encoding gene	15582	16205	+	Phage baseplate
ORF21	Protein encoding gene	16205	16624	+	Phage protein
ORF22	Protein encoding gene	16624	18093	+	Phage protein
ORF23	Protein encoding gene	18096	18953	+	Phage protein

ORF24	Protein encoding gene	18953	19255	+	Phage protein
ORF25	Protein encoding gene	19258	20424	+	Phage tail fiber
ORF26	Protein encoding gene	20476	22914	+	Phage tail fiber
ORF27	Protein encoding gene	22994	23188	+	Phage protein
ORF28	Protein encoding gene	23189	23560	+	Phage protein
ORF29	Protein encoding gene	24499	23600	-	Thymidylate synthase (EC 2.1.1.45)
ORF30	Protein encoding gene	25046	24501	-	Dihydrofolate reductase (EC 1.5.1.3)
ORF31	Protein encoding gene	25303	25043	-	Phage protein
ORF32	Protein encoding gene	25819	25304	-	Phage protein
ORF33	Protein encoding gene	26192	25833	-	Phage protein
ORF34	Protein encoding gene	26493	26194	-	Phage protein
ORF35	Protein encoding gene	26698	26486	-	Phage protein
ORF36	Protein encoding gene	27083	26700	-	Phage protein
ORF37	Protein encoding gene	28475	27372	-	DNA ligase, phage-associated
ORF38	Protein encoding gene	28668	28450	-	Phage protein
ORF39	Protein encoding gene	28871	28665	-	Phage protein
ORF40	Protein encoding gene	29135	28881	-	hypothetical protein
ORF41	Protein encoding gene	29280	29125	-	hypothetical protein
ORF42	Protein encoding gene	29510	29298	-	Phage protein
ORF43	Protein encoding gene	29716	29507	-	Phage protein
ORF44	Protein encoding gene	30301	29768	-	Phage protein, contains HNH endonuclease motif
ORF45	Protein encoding gene	33004	30305	-	DNA polymerase (EC 2.7.7.7), phage-associated
ORF46	Protein encoding gene	33261	33683	+	Phage protein
ORF47	Protein encoding gene	33685	34497	+	Phage minor tail protein
ORF48	Protein encoding gene	34559	35302	+	putative deoxynucleotide monophosphate kinase

ORF49	Protein encoding gene	35317	35511	+	Phage protein
ORF50	Protein encoding gene	35504	37489	+	Phage DNA primase/helicase
ORF51	Protein encoding gene	37742	37891	+	Phage protein
ORF52	Protein encoding gene	37966	38823	+	Phage protein
ORF53	Protein encoding gene	38886	39926	+	putative exodeoxyribonuclease
ORF54	Protein encoding gene	39916	40413	+	Phage protein
ORF55	Protein encoding gene	40435	40683	+	Phage protein
ORF56	Protein encoding gene	40659	41414	+	Phage protein
ORF57	Protein encoding gene	41395	41718	+	Phage protein
ORF58	Protein encoding gene	41711	42049	+	Phage protein
ORF59	Protein encoding gene	42096	44330	+	Ribonucleotide reductase of class Ia (aerobic), alpha subunit (EC 1.17.4.1)
ORF60	Protein encoding gene	44386	44643	+	Phage protein
ORF61	Protein encoding gene	44640	45713	+	Ribonucleotide reductase of class Ia (aerobic), beta subunit (EC 1.17.4.1)
ORF62	Protein encoding gene	45713	45955	+	Phage protein
ORF63	Protein encoding gene	45948	46154	+	Phage protein
ORF64	Protein encoding gene	46203	47402	+	Ribonucleotide reductase of class III (anaerobic), large subunit (EC 1.17.4.2)
ORF65	Protein encoding gene	47809	48663	+	Ribonucleotide reductase of class III (anaerobic), large subunit (EC 1.17.4.2)
ORF66	Protein encoding gene	48726	49061	+	hypothetical protein
ORF67	Protein encoding gene	49261	49656	+	Phage protein
ORF68	Protein encoding gene	49653	49952	+	Phage protein
ORF69	Protein encoding gene	49962	50447	+	Ribonucleotide reductase of class III (anaerobic), activating protein (EC 1.97.1.4)
ORF70	Protein encoding gene	50410	50787	+	Phage protein
ORF71	Protein encoding gene	50754	51011	+	Phage protein
ORF72	Protein encoding gene	51014	51334	+	Phage protein
ORF73	Protein encoding gene	51386	51919	+	Phage protein

ORF74	Protein encoding gene	51935	52816	+	Ribose-phosphate pyrophosphokinase (EC 2.7.6.1)
ORF75	Protein encoding gene	52862	54643	+	Nicotinamide phosphoribosyltransferase (EC 2.4.2.12)
ORF76	Protein encoding gene	54697	55032	+	Phage protein
ORF77	Protein encoding gene	55014	55190	+	Phage protein
ORF78	Protein encoding gene	55219	57585	+	Phage rIIA lysis inhibitor
ORF79	Protein encoding gene	57665	58774	+	Phage rIIB lysis inhibitor
ORF80	Protein encoding gene	58875	59423	+	Phage protein
ORF81	Protein encoding gene	59401	60096	+	Phage protein
ORF82	Protein encoding gene	60107	60571	+	Phage protein
ORF83	Protein encoding gene	60624	60971	+	Phage protein
ORF84	Protein encoding gene	60937	61134	+	Phage protein
ORF85	Protein encoding gene	61131	61400	+	Phage protein
ORF86	Protein encoding gene	61403	61729	+	Phage protein
ORF87	Protein encoding gene	61704	61931	+	Phage protein
ORF88	Protein encoding gene	61924	62124	+	Phage protein
ORF89	Protein encoding gene	62125	62910	+	Phage protein
ORF90	Protein encoding gene	63431	63637	+	Phage protein
ORF91	Protein encoding gene	63727	64107	+	Phage protein
ORF92	Protein encoding gene	64196	64486	+	Phage protein
ORF93	Protein encoding gene	64577	65089	+	Phage protein
ORF94	Protein encoding gene	65181	65477	+	Phage protein
ORF95	Protein encoding gene	65471	65803	+	Phage protein
ORF96	Protein encoding gene	65894	66166	+	Phage protein
ORF97	Protein encoding gene	66252	66656	+	Phage protein
ORF98	Protein encoding gene	66758	66961	+	Phage protein

ORF99	Protein encoding gene	67054	67596	+	Phage protein
ORF100	Protein encoding gene	67663	67896	+	Phage protein
ORF101	Protein encoding gene	67984	68325	+	Phage protein
ORF102	Protein encoding gene	68549	68797	+	Phage protein
ORF103	Protein encoding gene	69553	69666	+	hypothetical protein
ORF104	Protein encoding gene	71085	70537	-	Phage protein
ORF105	Protein encoding gene	71563	71099	-	Phage protein
ORF106	Protein encoding gene	71981	71553	-	Phage protein
ORF107	Protein encoding gene	72257	71985	-	hypothetical protein
ORF108	Protein encoding gene	72535	72311	-	Phage protein
ORF109	Protein encoding gene	72630	72535	-	hypothetical protein
ORF110	Protein encoding gene	73115	72612	-	Phage protein
ORF111	Protein encoding gene	73330	73112	-	Phage protein
ORF112	Protein encoding gene	73995	73414	-	Phage protein
ORF113	Protein encoding gene	74339	73995	-	Phage protein
ORF114	Protein encoding gene	74625	74332	-	Phage protein
ORF115	Protein encoding gene	75020	74625	-	Phage protein
ORF116	Protein encoding gene	75411	75013	-	Phage protein
ORF117	Protein encoding gene	75929	75465	-	Phage lysin (EC 3.2.1.17)
ORF118	Protein encoding gene	76561	75929	-	Phage protein
ORF119	Protein encoding gene	76929	76558	-	Phage protein
ORF120	Protein encoding gene	77567	76977	-	Phage protein
ORF121	Protein encoding gene	77794	77561	-	Phage protein
ORF122	Protein encoding gene	77937	77776	-	Phage protein
ORF123	Protein encoding gene	78187	78558	+	Phage protein

ORF124	Protein encoding gene	78640	80055	+	Phage protein
ORF125	Protein encoding gene	80830	81258	+	hypothetical protein
ORF126	Protein encoding gene	83583	84140	+	Phage protein
ORF127	Protein encoding gene	84592	85326	+	Phage protein
ORF128	Protein encoding gene	85723	85923	+	Phage protein
ORF129	RNA	80221	80294	+	tRNA-Pro-TGG
ORF130	RNA	80305	80379	+	tRNA-Glu-TTC
ORF131	RNA	80720	80804	+	tRNA-Pseudo-GTA
ORF132	RNA	81264	81336	+	tRNA-Asp-GTC
ORF133	RNA	81769	81841	+	tRNA-Lys-TTT
ORF134	RNA	81927	81999	+	tRNA-Ile-GAT
ORF135	RNA	82250	82323	+	tRNA-Arg-TCT
ORF136	RNA	82908	82981	+	tRNA-Leu-TAG
ORF137	RNA	82992	83064	+	tRNA-Lys-CTT
ORF138	RNA	83074	83146	+	tRNA-Ala-TGC
ORF139	RNA	83156	83227	+	tRNA-Gly-TCC
ORF140	RNA	83238	83311	+	tRNA-Thr-TGT
ORF141	RNA	83410	83481	+	tRNA-Val-TAC
ORF142	RNA	83486	83560	+	tRNA-Leu-CAA
ORF143	RNA	84149	84221	+	tRNA-Gln-TTG
ORF144	RNA	84227	84302	+	tRNA-Leu-TAA
ORF145	RNA	84311	84383	+	tRNA-Gln-CTG
ORF146	RNA	84418	84490	+	tRNA-His-GTG
ORF147	RNA	84500	84572	+	tRNA-Phe-GAA
ORF148	RNA	85425	85497	+	tRNA-Cys-GCA
